# The Left Hand Second to Fourth Digit Ratio (2D:4D) Does Not Discriminate World-Class Female Gymnasts from Age Matched Sedentary Girls

**DOI:** 10.1371/journal.pone.0040270

**Published:** 2012-06-29

**Authors:** Maarten W. Peeters, Albrecht L. Claessens

**Affiliations:** Department of Kinesiology, KU Leuven, Leuven, Belgium; University of South Australia, Australia

## Abstract

**Introduction:**

The second to fourth-digit-ratio (2D:4D), a putative marker of prenatal androgen action and a sexually dimorphic trait, has been suggested to be related with sports performance, although results are not univocal. If this relation exists, it is most likely to be detected by comparing extreme groups on the continuum of sports performance.

**Methods:**

In this study the 2D:4D ratio of world-class elite female artistic gymnasts (n = 129), competing at the 1987 Rotterdam World-Championships was compared to the 2D:4D ratio of sedentary age-matched sedentary girls (n = 129), alongside with other anthropometric characteristics including other sexually dimorphic traits such as an androgyny index (Bayer & Bayley) and Heath-Carter somatotype components (endomorphy, mesomorphy, ectomorphy) using AN(C)OVA. 2D:4D was measured on X-rays of the left hand.

**Results:**

Left hand 2D:4D digit ratio in world class elite female gymnasts (0.921±0.020) did not differ significantly from 2D:4D in age-matched sedentary girls (0.924±0.018), either with or without inclusion of potentially confounding covariates such as skeletal age, height, weight, somatotype components or androgyny index. Height (161.9±6.4 cm vs 155.4±6.6 cm p<0.01), weight (53.9±7.6 kg vs 46.2 6.3 kg p<0.01), BMI (20.51±2.41 kg/m^2^ vs 19.05±1.56 kg/m^2^), skeletal age (15.2±1.1 y vs 14.5±1.2 y p>0.01), somatotype components (4.0/3.0/2.9 vs 1.7/3.7/3.2 for endomorphy (p<0.01), mesomorphy (p<0.01) and ectomorphy (p<0.05) respectively) all differed significantly between sedentary girls and elite gymnasts. As expressed by the androgyny index, gymnasts have, on average, broader shoulders relative to their hips, compared to the reference sample. Correlations between the 2D:4D ratio and chronological age, skeletal age, and the anthropometric characteristics are low and not significant.

**Conclusion:**

Although other anthropometric characteristics of sexual dimorphism were significantly different between the two samples, the present study cannot discriminate sedentary girls from world-class female gymnasts by means of the left hand 2D:4D ratio.

## Introduction

Although the ratio of the length of the second (index) finger to the length of the fourth (ring) finger (2D:4D) has been the subject of much recent research [1]–[11] the sexually dimorphic nature of this ratio has been reported for over more than 60 years [12]. The ratio represents an individual difference variable putatively related to prenatal gonadal hormonal exposure. A lower 2D:4D is indicative of relatively higher prenatal testosterone than estrogen levels, which means that men may have, on average, lower digit ratios than women [13]. Several studies indicate that the digit ratio is related with psychological characteristics like assertiveness and aggression [13], with the onset of menarche [4], [14], with homosexuality [13], with success among financial traders [15], and with neck circumference, indicating that in men a higher 2D:4D ratio is associated with greater risk of obesity and heart disease [16].

A number of studies gave investigated the associations between 2D:4D and sporting ability or physical fitness [1]–[3], [5], [7], [9], [11], [17]–[24]. The hypotheses set up in these studies were that a lower digit ratio is related to better sport abilities or better motor performance, in both males and females. Overall, results of the studies are not consistent and in part difficult to compare because of the use of different procedures for measuring the digit lengths. In some studies where both sexes are included the relation is found in both men and women [3], [18], [22], although in the study of Hönekopp et al. [18] the relation in women was significant only for the left hand 2D:4D and not for the right hand 2D:4D and vice versa in men. Other studies found a significant relation in men but not in women [7] and vice-versa [11]. Fink et al [17] reported a relation between 2D:4D and hand-grip strength in men, but this was not replicated in men [24] nor found in women [23], [24]. A number of studies found the relationship between sporting ability and digit ratio in both right and left 2D:4D [1], [7] in right hand 2D:4D but not in left hand 2D:4D [2], [11], [17] or vice versa [21] with the mean of right and left 2D:4D [9] or neither with right nor left 2D:4D [5], [24], but only with the difference between right and left 2D:4D [5]. In a meta-analysis Hönekopp and Schuster [25] reported that for the association between 2D:4D digit ratio and athletic prowess neither hand outpredicted the other. Also, in many studies sporting abilities were not measured objectively, but based on information reported by the subjects themselves [9], [22]. In addition, many of the published studies on sporting ability and 2D:4D focused on physical fitness or sport ability in general or a combination of sports and not on one specific sport, and studies including both sedentary subjects and objectively assessed elite-status athletes are limited [1].

To our knowledge, a study that focuses on a large sample of elite female gymnasts to investigate the association between the digit ratio and artistic gymnastic performance is not available. If indeed there is an association between digit ratio and sporting ability it is most likely to be detected by comparing extreme groups on the continuum of sports performance. The main aim of this study therefore was to compare the 2D:4D ratio of world-class elite female artistic gymnasts with the 2D:4D ratio of sedentary age-matched reference girls. It is hypothesized that the group of elite gymnasts have a significant lower digit ratio compared to the reference group.

## Materials and Methods

### Samples

#### Gymnast sample

The sample (n = 129) of elite female gymnasts was a sub-sample of the participants of the 24^th^ World Championships Artistic Gymnastics held at Rotterdam, The Netherlands, in 1987, which has been previously described [26]–[28]. Because the reference sample (see below) consisted of Caucasian girls up to 18.5 yrs old, and 2D:4D varies with ethnicity [13] we selected only gymnasts of Caucasian ethnicity and ages up to 18.5 yrs. Their chronological age varied from 13.2 to 18.5 years, with a mean age of 16.1±1.2 years. The study in the frame of which this dataset was collected was approved by the Medical Ethics Committee of the Institute of Physical Education of the Catholic University of Leuven and by the Medical committee of the Fédération Internationale de Gymnastique (FIG). Prior to the championships, the national gymnastics federation of each participating country was contacted about the study by the Medical committee of the FIG. Subsequently the delegation chiefs, coaches, and/or medical officers as legal guardians for the minors during the competition, were personally approached at the competition site for permission to contact individual athletes and to perform the measurements in their presence. Only if the delegation chief, coach and/or medical officer provided permission, the individual gymnasts were contacted, informed about the study and provided verbal consent if they wanted to cooperate. The procedure for contacting the delegation chiefs, coaches and medical officers and the individual athletes and obtaining verbal consent was approved by the Medical Ethics Committee of the Institute of Physical Education of the Catholic University of Leuven and by the Medical committee of the FIG [28]. All data was analyzed anonymously.

#### Reference sample

Girls from the Leuven Growth Study of Flemish Girls (LGSFG) [29] were used as the reference sample. The LGSFG (n = 9414 girls between 6–18 years) was a representative sample of the Flemish population in the academic year of 1979–1980. The sampling was done with the guidance of the Statistical Service of the Belgian Ministry of education using a multistage procedure. In the first stage a proportionate stratified sample with schools as the primary sampling cluster was selected. This sample included 43 primary schools and 45 secondary schools. In the second stage, all students in a single classroom at each grade level were selected within each school [29]. From this study a sample of 129 age-matched girls was selected. The matching by chronological age was accurate within 0.1 year. Besides this, only reference girls with the following conditions were selected: (1) girls for whom an X-ray of the left hand was available; and (2) girls must be sedentary, i.e. not practice any sports apart from the mandatory 2 hours/week of physical education classes in school, to be sure of a significant difference in sporting ability between the two samples. The mean chronological age of the selected girls was 16.1±1.3 years, varying from 13.2 to 18.4 years. The Leuven growth study of Flemish Girls was approved by the Medical Ethics Committee of the Institute of Physical Education of the Catholic University of Leuven. Written informed consent was given by the school authorities at the national and local levels, and by the parents of the children [29].

### Anthropometric dimensions

The following measurements were taken: weight; height; biacromial and bicristal breadths; humerus and femur widths; biceps and calf girths; and triceps, subscapular, supraspinale, and calf skinfolds. All bilateral measurements were taken on the left side of the body. Body mass index (BMI) was calculated as weight (kg) / height (m^2^). All measurements were taken by well-trained observers according to the measuring procedures as described by Claessens et al. [30].

### Body ratio of sexual dimorphism

The *androgyny index*: the Bayer-Bayley ratio (Androgyny Index, IANDR) [31] relates the breadth of the hips (lower trunk, bicristal breadths) to that of the shoulders (upper trunk, biacromial breadth).
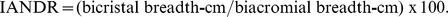



On average the ratio is higher in girls than in boys at virtually all ages during childhood and adolescence, and this difference persists into adulthood.

This index is a useful indicator of sex differences in the proportional relationship of the shoulders and hips [31]–[33].

### Somatotype

The three somatotype components endomorphy, mesomorphy and ectomorphy were anthropometrically determined according to the Heath-Carter technique. The Heath-Carter anthropometric somatotype is calculated from 10 dimensions: weight; height; triceps, subscapular, supraspinale, and calf skinfolds; humerus and femur widths; and biceps and calf girths. Endomorphy is derived from the sum of three skinfolds (triceps, subscapular, supraspinale) adjusted for height. This component describes the relative degree of fatness of the body. Mesomorphy is derived from humerus and femur widths, biceps and calf circumferences corrected for the triceps and calf skinfolds respectively, and height. The four limb measurements are adjusted for height. This component expresses the relative degree of muscle, bone and connective tissue. Ectomorphy is based on the somatotype ponderal index: height (cm) divided by weight (kg)^1/3^. This component characterizes the degree of linearity, slenderness, and fragility of body build, with poor muscular development, and a predominance of surface area over body mass. For a detailed description how the three components were calculated reference is given to Claessens et al. [32].

### Skeletal age

An X-ray of the left hand and wrist of each subject was taken for the assessment of skeletal maturity. An Elinax 60 (62 kV, 15mA) assembled in a portable apparatus was used. Skeletal age (Skel.age) was estimated using the radius-ulna-short bone protocol in the Tanner-Whitehouse II method. With this method, the maturity status of the radius, ulna, the first, third, and fifth metacarpals, and phalanges of the first, third and fifth fingers are rated, the bone-specific scores are summed into a maturity score, and the maturity score is converted to a skeletal age. For more detailed information reference is given to Claessens et al. [32].

### Measuring 2D and 4D lengths

#### Measuring procedure

Radiographs from the left hand of all the subjects of the two samples were available to measure and calculate 2D:4D. The lengths of the second and fourth finger were measured from the proximal end of the proximal phalanx to the distal tip of the distal phalanx using a caliper accurate to 0.1 mm (John Bull British Indicators LtD, England). Digit lengths of each sample were measured by two raters. The mean of the two raters was taken as the final measurement.

#### Reliability study

Before measuring all X-rays, a reliability study was conducted by three different raters. This study consisted of the measurement of thirty left-hand X-rays twice by each rater in a test and re-test manner. Interobserver measurement repeatabilities for 2D:4D ratios were assessed with intraclass correlation coefficients (ICC). The ICC for interrater reliability showed a reliability of 0.98. Anova did not show any significant difference for 2D:4D between the raters. The technical error of measurement for all raters was <0.001 for 2D:4D. It therefore can be concluded that all measurements of 2D:4D were measured reliable.

### Statistical analyses

Differences in 2D:4D and in anthropometric characteristics between the sample of elite gymnasts and the reference group were analyzed by means of a two-tailed t-test. If a standard deviation for the digit ratio of 0.02 is assumed and a 0.01 difference in digit ratio between the two samples is to be detected, the statistical power of the present study is 0.98 when alpha = 0.05. An ANCOVA (Proc GLM, SAS 9.1.3) was used to compare the two samples for each variable with skeletal age as the covariate. The relationship between 2D:4D ratio and anthropometric characteristics for each sample were calculated by Pearson Product Moment correlation coefficients. The Statistical Analysis System program 9.1.3 (SAS Institute, Cary, NC, USA) was used to analyze the data.

## Results

Descriptive statistics (mean ± SD) of all variables for both samples are given in Table 1.

**Table 1:** Descriptive statistics.

	Reference girls (n = 129)	Gymnasts (n = 129)	t-value
	Mean ± SD	Mean ± SD	
Chron. age (yrs)	16.1±1.3	16.1±1.2	0.32
Skel. Age (yrs)	15.2±1.1	14.5±1.2	4.89**
2D:4D	0.924±0.018	0.921±0.020	1.28
Height (cm)	161.9±6.4	155.4±6.6	8.17**
Weight (kg)	53.9±7.6	46.2±6.3	8.83**
BMI (kg/m^2^)	20.51±2.42	19.05±1.56	5.75**
Endomorphy	4.0±1.1	1.7±0.6	19.90**
Mesomorphy	3.0±0.9	3.7±0.6	−6.82**
Ectomorphy	2.9±1.3	3.2±0.8	−1.98*
IANDR	76.39±4.29	73.11±4.28	6.16**

IANDR: androgyny index; **p<0.01; *p<0.05.

Mean chronological ages of both samples do not differ significantly, as expected, whereas skeletal age of the gymnasts' sample is significantly lower compared to the skeletal age of the reference girls, 14.5±1.2 and 15.2±1.1 respectively. Height, weight and BMI of the elite gymnasts are significantly lower compared to the reference sample. Also, marked differences in somatotype between the two groups of girls can be observed. Gymnasts are more mesomorph and ectomorph, and less endomorph compared to reference girls, with mean somatotypes of 1.7/3.7/3.2 and 4.0/3.0/2.9 respectively. As expressed by IANDR, gymnasts have, on average, broader shoulders relative to their hips, compared to the reference sample. No significant difference between both groups for the digit ratio can be observed, 0.924±0.018 and 0.921±0.020 for the reference girls and gymnasts respectively. Because of the significant difference in skeletal age between the two samples, skeletal age was added as a covariate in an ANCOVA. The adjusted means of the 2D:4D ratio and of the anthropometric characteristics are given in Table 2.

**Table 2:** Adjusted means of 2D:4D and of the anthropometric characteristics (ANCOVA with skeletal age as co-variate).

	F-ratio	LSMEAN ref. girls	LSMEAN gymnasts
		(n = 129)	(n = 129)
2D:4D	1.31	0.924	0.921
Height (cm)	46.82**	161.3	155.8
Weight kg)	49.32**	52.7	47.3
BMI (kg/m^2^)	12.13**	20.19	19.39
Endomorphy	334.48**	3.9	1.9
Mesomorphy	60.14**	2.9	3.7
Ectomorphy	0.02	3.1	3.1
IANDR	26.16**	76.15	73.32

IANDR: androgyny index; **p≤0.01.

Although all variables were adjusted for skeletal age, it is observed that all the adjusted mean differences for the anthropometric variables between the two samples are still significant, with the exception of the ectomorphy component. Adjusted mean values for the 2D:4D ratio for both samples did not change as compared to the absolute means. Results similar to those reported in table 2 were obtained for the 2D:4D when covarying for the anthropometric measurements and indices (results not shown).

Correlations between the 2D:4D ratio on the one hand and chronological and skeletal ages, and the anthropometric characteristics on the other hand are low and not significant (p>0.05), ranging from r = 0.11 (with skeletal age) to r = −0.16 (with height) in the reference sample, and from r = 0.15 (with height) to r = −0.10 (with mesomorphy) in the gymnasts sample.

## Discussion

The present study shows that left hand 2D:4D digit ratio in world-class elite gymnasts is not different from left hand 2D:4D digit ratio in age matched sedentary girls. However both groups are significantly different for a number of objectively measured anthropometric characteristics which shows that the gymnast do have a more ‘masculine' body morphology. For the gymnasts' sample, we hypothesized a lower 2D:4D ratio, compared to the value found for the reference sample, but no significant difference between both mean ratios was observed ( 0.924±0.018 and 0.921±0.020 for the reference girls and gymnasts respectively). So the 2D:4D ratio, on average, may not be a discriminating factor for artistic gymnastic performance. This is consistent with the findings of a study by Paul et al. [9] about the relationship between 2D:4D and sporting ability across a range of 12 sports in a sample of 607 female participants. The subjects were asked to give the highest competitive level in their sport activity on a five-point scale, with ‘social participation only’ as the lowest level ( = 1) and ‘national level’ ( = 5) as the highest level. Measurements of the digit lengths were done on X-rays. The overall age-adjusted level achieved in any sport was significantly negatively associated with mean 2D:4D. But, when analyzed separately, mean digit ratio was only significantly associated with running level. There was no significant relationship between 2D:4D and the level in a subsample of female gymnasts. A mean left-hand 2D:4D ratio of 0.93±0.02 was found, which is similar to the value of 0.92 found in the present study.

The findings of our study are consistent with the findings of Paul *et al*. [9] for gymnasts and with those on other measures of sporting abilities in women such as ergometer rowing performance [7], national or international fencing ranking with left 2D:4D [2], [11], and handgrip strength [23], [24]. However they are in contrast with studies in which significant relationships between 2D:4D and sporting ability in females were observed with the dominant hand 2D:4D [3], the left hand 2D:4D [21], or the right hand 2D:4D [2], [11], [22] although none of these studies contrasted world-class elite athletes with sedentary reference samples.

The 2D:4D ratio is moderately related to performance in endurance running of young adults with correlations varying from r = 0.30 to r = 0.50 [19]. In contrast little evidence is found for the relationship between 2D:4D and acceleration and strength. Correlation coefficients between 2D:4D and sprinting speed in boys were weak (r = 0.15; p = 0.02) in a study by Manning & Hill [20]. van Anders (2007) found no significant association between 2D:4D and grip-strength in a sample of 99 women (mean age 23.76±5.66 years) and neither did Gallup et al. [24]. This suggests that the widespread relationship between 2D:4D and sport performance may have more to do with aerobic efficiency than with strength and acceleration [20] although speed is also crucial in fencing [2], [11]. It is possible that strength and acceleration are two more important modifying determinants in artistic gymnastic performance, compared to aerobic capacity and hence our results are consistent with the lack of evidence for an association between handgrip strength and 2D:4D in women [23], [24]. The physical capacities that serve as a basis for gymnastic talent are speed, quickness, flexibility and strength [34]. Gymnasts do not perform competitive routines longer than 90 seconds. Therefore, the oxidative energy system is probably not a dominant energy system for gymnastics. The anaerobic dominance of gymnastic performance is supported by several studies [35].

The androgyny index and somatotype are, just like the 2D:4D ratio, determinants of sexual dimorphism. Men have on average a lower 2D:4D ratio [13], a higher mesomorphy component, a lower endomorphy component, and a lower value for the androgyny index, compared to women [31]–[33]. As expressed in several previous studies [27] elite female gymnasts demonstrate a more ‘masculine’ body morphology compared to age-related reference girls. This is also demonstrated in the present study. The sample of elite gymnasts has significant lower IANDR, which means that the gymnasts have on average broader shoulders relative to their hips, compared to the reference girls, 73.11 and 76.39 respectively for the Bayer-Bayley index. When looking at the body as a ‘Gestalt’, a significant difference was found in somatotype between the two samples. Gymnasts demonstrated on average a somatotype of 1.7/3.7/3.2 compared to an average somatotype of 4.0/3.0/2.9 of the reference sample. Elite gymnasts are characterized by an ecto-mesomorphic somatotype whereas reference girls are characterized as meso-endomorphic. Mesomorphy is characterized by the predominance of muscle, bone and connective tissue, whereas endomorphy describes the degree of roundness and fatness of the body. Although both samples can be sexually discriminated on the basis of anthropometric characteristics, the left hand 2D:4D ratio does not.

In addition, when testosterone is negatively related to 2D:4D [13], it is expected that more ‘male’ forms of 2D:4D would correlate with more ‘male’ forms of anthropometric characteristics like androgyny indices and somatotype. Some previous studies have investigated the relationship between 2D:4D and anthropometric characteristics. Fink et al. [36] investigated the relationship between 2D:4D with body mass index, waist-to-hip ratio and waist-to-chest ratio. Some evidence was found that 2D:4D also correlates with indices of sexually dimorphic traits of the human body. Body fat distribution was in that study measured by the waist-to-hip ratio. However, no significant associations were observed for male and female 2D:4D and the waist-to-hip ratio. In females, no significant relationship between body mass index and 2D:4D was found. A higher value of 2D:4D correlated significantly with a lower value of the waist-to-chest ratio. This is consistent with the literature as oestrogens should largely influence chest circumference in females. In a study by Gallup et al. [24] the relationship between handgrip strength and three measures of body morphology (shoulder-to-hip ratio, waist-to-hip ratio, 2D:4D) was investigated in a sample of 82 male and 61 female college students. A significant positive relationship between 2D:4D and waist-to-hip ratio in females could be observed. This however is contrary to what might be hypothesized if both traits indeed are sexually dimorphic: a lower waist-to-hip ratio reflects a female fat – distribution pattern while a lower 2D:4D reflects the more masculine phenotype of the digit ratio. Hence the results of Gallup et al. [24] suggest that a lower, andtherefore more masculine, digit ratio is related to amore feminine fat patterning as reflected by the lower waist-to-hip ratio. Because mostly different body ratios of sexual dimorphism were used in the literature compared to the one used in the present study, comparison of results remains difficult. Since the IANDR used in the present study is based in essence on bone-measurements, it might have been expected that a relation between this sexually dimorphic trait and the digit ratio was more likely than that with circumference measurements related to fat patterning. Although most of the anthropometric characteristics used in the present study were discriminating factors between the gymnasts and the reference samples, no significant correlations were observed between 2D:4D and any of these anthropometric characteristics. Therefore adding them as covariates in an Ancova did not alter the results (table 2), not even for height (results not shown), the only trait that might be considered to be more masculine in the reference sample and therefore might have potentially ‘masked’ a difference in 2D:4D digit ratio between both groups.

The present study is to our knowledge the first study that investigated the relationship between 2D:4D in a large sample of female gymnasts of world-class level (n = 129), by comparing their 2D:4D ratio with that of an age-matched reference sample (n = 129) of sedentary girls. The high level of the gymnasts' sample is based on the fact that all gymnasts participated at the World Championships Artistic Gymnastics held in Rotterdam, 1987 [26], [27]. The reference sample consisted of girls who were all sedentary. There was thus an obvious difference in sporting ability between both groups. In many studies about the relationship between sporting ability and 2D:4D, the level of sporting ability was based on information reported by the subjects themselves. Furthermore, in the present study, all anthropometric variables were measured objectively; whereas in a lot of previous studies data (e.g. height and weight) were reported.

It may be noted that the mean value of 0.924 (SD = 0.018) for the 2D:4D ratio found for the reference sample is quite low compared to e.g. 2D:4D ratio from a sample of 531 females which had a mean 2D:4D ratio of 1.00 (SD = 0.03) [13]. This low value is however not entirely unexpected since, although this was not verified in the present study, Manning et al. [37] had already observed that mean radiograph-derived 2D:4D showed lower ratios than those from photocopies and showed less sexual dimorphism although they are significantly correlated. Therefore a possible explanation for this low mean value observed for our reference sample is that the digit lengths were measured on X-rays in contrast to the measuring procedures used in many other studies in which mostly digit lengths were measured on photocopies, printed scans or directly from the hand. Previous studies have compared different methods of digit ratio measurement [13], [38]–[40]. The fact that measurements on X-rays seem to yield lower digit ratios may be partially explained by the fact that measurements made on soft tissue or images of the soft tissue on the hand are taken approximately halfway along the proximal phalanx whereas bone measurements begin at the proximal end of the phalanx [13]. This hypothesis has not been subject to research yet but could possibly provide an explanation and may lead to standardization for the measurement of the 2D:4D ratio. At the same time when measuring on photocopies, printed scans or directly on the hand, the soft tissue is also measured whereas when measuring finger lengths on X-rays only the bone length is recorded. In a study of Paul et al. [41], concerning the heritability of the 2D:4D ratio, measurements of the finger lengths were also made on X-rays. In a sample of 456 female twin pairs a mean 2D:4D ratio of 0.92 (SD = 0.001) was observed for both hands, which is similar to the 2D:4D ratio found in the present study. Although it was not formally tested in the present study, the analogy with findings from the literature regarding different methods of determining finger lengths, it seems plausible that the low 2D:4D ratio of 0.924 found in our reference sample is the result of the fact that the measurements were done on X-rays.

A possible limitation of our study is that all measurements of the digit lengths were taken on the left hand. In most studies the digit lengths were taken on the right hand to calculate 2D:4D. This is based on the fact that results of previous studies have suggested that sex differences in 2D:4D and correlations of 2D:4D with target traits are more pronounced for the right hand than for the left hand [6] Testosterone-dependent physical traits tend to be more strongly expressed on the right side of the body compared to the left side [13], however a meta-analyses focusing specifically on the relation between the 2D:4D digit ratio and athletic prowess demonstrated that neither hand outpredicts the other. [25].

In conclusion, although other anthropometric characteristics of sexual dimorphism were significantly different between the two samples, the present study cannot discriminate sedentary girls from world-class gymnasts by the left hand 2D:4D ratio. Furthermore, no significant correlations were found between left hand 2D:4D ratios and anthropometric and age characteristics, both chronological and skeletal.
